# Supplementary material for: Seroincidence of SARS-CoV-2 infection prior to and during the rollout of vaccines in a community-based prospective cohort of U.S. adults
Source: Sci Rep. 2024 Jan 5;14:644. doi: 10.1038/s41598-023-51029-9 (PMC10770061; doi:10.1038/s41598-023-51029-9)
Supplement: Supplementary file 1 — Supplementary Information. [file 41598_2023_51029_MOESM1_ESM.docx]

**Seroincidence of SARS-CoV-2 infection prior to and during the rollout of vaccines in a community-based prospective cohort of U.S. adults [Supplementary Material]**

**STATISTICAL APPENDIX**

*LASSO.* The study samples of 3,421 and 2,735 participants for the pre-vaccine/wild type and post-vaccine variant eras respectively were split randomly into equally sized training and test data sets. A grouped LASSO regression was fit using training data and 10-fold cross validation was used to obtain the minimum value of lambda (the tuning parameter). The variables included in the LASSO model were household crowding, having children in the household, confirmed COVID-19 case in a household member, attending mass gatherings, indoor dining in a restaurant/bar, outdoor dining in a restaurant/bar, visiting places of worship, visiting public park or pool, gathering with groups of 10 or more indoors and/or outdoors, mask use while grocery shopping, mask use while visiting non-household members, mask use indoors in gym/salons, mask use indoors at work, mask use outdoors, using public transit, recent air travel, alcohol use, and substance use. Using the minimum lambda value, a grouped LASSO model was run on the entire dataset (training + test) to obtain factors that best predicted seroconversion. The LASSO model selected having a confirmed COVID-19 case in a household member, indoor dining in a bar/restaurant, gathering with groups of 10 or more outdoors, and mask use indoors in salons/gyms as the most predictive of seroconversion in our cohort. A logistic regression model was fitted using these variables selected by LASSO regression with seroconversion (Y/N) as the outcome. Coefficients of the variables were multiplied by 10 and rounded up to the nearest integer to create a score associated with that variable/risk factor. If a participant engaged in a given risk factor, they were assigned the score associated with that risk factor. Scores were then summed across risk factors, normalized between 0 and 100, and a total composite risk score was created for each participant.
